# Sustainability in gynecology and obstetrics ‐ now or never!

**DOI:** 10.1111/aogs.14457

**Published:** 2022-09-30

**Authors:** Wouter J. K. Hehenkamp, Martin Rudnicki

**Affiliations:** ^1^ Department of Obstetrics and Gynecology and Center for Sustainable Healthcare Amsterdam University Medical Center Amsterdam The Netherlands; ^2^ Department of Obstetrics and Gynecology Odense University Hospital Odense Denmark

Climate change has become increasingly apparent in the last decade. The Intergovernmental Panel on Climate Change (IPCC) report of 2021 was very clear stating that climate change is the biggest threat to human health and is caused by human action.^1^ Barack Obama put it into words: ‘*we are the first generation to feel the effects of climate change and the last generation that can do something about it’*. Therefore, sustainability should be our top priority in order to save mankind from health disasters such as water shortage, increasing hunger in third world countries and possibly even pandemics such as the corona virus.

Even though no one is ‘against’ fighting climate change, making green choices will inevitably affect our freedom to live. So far not much has changed. After the corona crisis the number of international flights is almost back to normal and meat consumption has never been higher. Thus, we must ask everyone to reconsider their consumption. Accordingly, the healthcare sector needs to reconsider its boundaries when it comes to environmental impact. The healthcare sector is responsible for the emission of 7% of all carbon gasses in the Netherlands.^2^

Recently, the Dutch healthcare system published a strategy (i.e., the carbon footprint) through ‘The Green Deal Sustainable Healthcare for a Healthy Future’.^3^ This strategy outlines an ambitious target to become a net zero healthcare system in 2050. This plan sounds fantastic, but in the Netherlands no big changes have been put in motion and the carbon reduction goal will not be met if we do not make substantial changes quickly. In Denmark, it has been decided that the carbon footprint must be reduced from 217.000 tons CO2 to 55.000 tons carbon before 2030.^4^ This includes more climate friendly buildings, optimized transportation, and a circular economy without waste. However, this needs also to be implemented and like the Netherlands not nearly enough has happened. Furthermore, both strategies are setting the overall goals, but do not specifically describe how to meet the goals.

One way of looking at carbon emission reduction strategies is by the ‘levels of circularity’. Although there has been a debate how to define circular economy, in general it means that all materials should be used in such a way that they can be cycled indefinitely. This, however, implies several problems. Thus, it has to happen on a time‐scale which is relevant. Nuclear waste may need thousands of years to be potentially safe. Further, if materials should be cycled then preferably intact as a product, but then as components, and finally recycled back to raw materials. But looking deeper into this, more complexity exists depending on the context.

**FIGURE 1:**
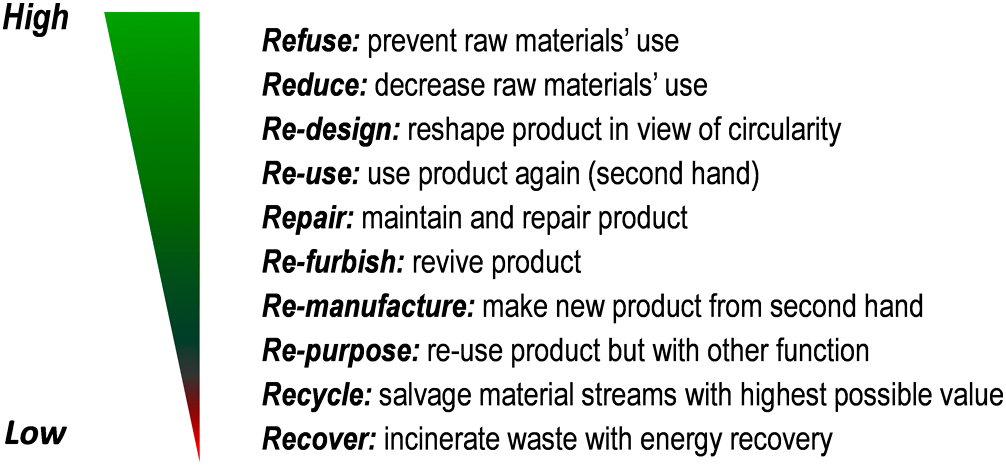
Levels of circularity

Ten R‐words demonstrate what has the highest impact on Carbon emission reduction^5^ (Figure 1). The highest impact is achieved by Refusing or Reducing using materials. Recycling is probably the most applied green action in hospitals but has a very low impact on carbon emission. When we want to make bigger changes, we need to rethink our strategy and stop applying healthcare that is inappropriate (Refuse). Applying ‘appropriate care’ is characterized by evidence‐based care, clinical expertise, patient‐centeredness, resource use, and equity.^6^

Another way of evaluating carbon emission is by life‐cycle analysis. This is a standardized, science‐based tool for quantifying the impact to assess lifetime environmental influence of the product/buildings etc. It takes all steps into consideration and gives the health care system an option to identify products with a low impact on carbon emission, but also other dimensions can be considered such as ozone depletion, impact on human health etc. Finally, life‐cycle‐analysis can be trusted since they are based on international standards. Such analysis could include an estimation of carbon emission from the use of disposable instruments,^7^ calculation on the impact of different surgical procedures (laparoscopic vs robotic assisted surgery) and if needed discussion of the need of such procedures which in many ways could be replaced by more simple non‐surgical interventions.^8^ Thus, so far there are no incentives for being extra‐careful but introducing Life Cycle Analysis may have an impact on the individual focus on waste handling. Further, introduction of knowledge regarding carbon emission data related to specific instruments, procedures etc may be followed by use with caution.

Sustainability can also be described by use of the three P's which include People, Profit and Planet. These P's should be in balance with one another. Thus, without a healthy Planet, there is no future for the People, and making Profit should never be at the expense of the People and/or the Planet. This also demonstrates that saving the planet is not only about reducing carbon concentrations, but also about saving eco‐systems, using resources wisely, preventing chemicals to leak into surface water etc (i.e. environmental impact). In healthcare we are not aware enough of the consequences of our action for the planet. We should gain more insight in the effect of different treatments on these outcomes.

Besides this, we need awareness of the caregivers and their management. Starting small green projects in a hospital (no more plastic cups, recycling, re‐using materials etc) may increase this awareness and provide support for the bigger sustainable choices. But also, we should look at our own actions: should we fly all over the world to visit international conferences?

The climate crisis may be the biggest problem facing us this century. Doing nothing is simply not an option. As doctors we serve as role model to the next generation of doctors. It is our responsibility to lead by example. We should use public resources as if they were our own, and as if we would have to fork out money every time we used a resource.
